# Photorespiratory Bypasses Lead to Increased Growth in *Arabidopsis thaliana*: Are Predictions Consistent with Experimental Evidence?

**DOI:** 10.3389/fbioe.2016.00031

**Published:** 2016-04-07

**Authors:** Georg Basler, Anika Küken, Alisdair R. Fernie, Zoran Nikoloski

**Affiliations:** ^1^Department of Chemical and Biomolecular Engineering, University of California Berkeley, Berkeley, CA, USA; ^2^Department of Environmental Protection, Estación Experimental del Zaidín CSIC, Granada, Spain; ^3^Systems Biology and Mathematical Modeling Group, Max Planck Institute of Molecular Plant Physiology, Potsdam-Golm, Germany; ^4^Central Metabolism Group, Max Planck Institute of Molecular Plant Physiology, Potsdam-Golm, Germany

**Keywords:** flux balance analysis, *Arabidopsis thaliana*, photorespiration, metabolic bypasses, metabolic engineering, crop optimization

## Abstract

Arguably, the biggest challenge of modern plant systems biology lies in predicting the performance of plant species, and crops in particular, upon different intracellular and external perturbations. Recently, an increased growth of *Arabidopsis thaliana* plants was achieved by introducing two different photorespiratory bypasses via metabolic engineering. Here, we investigate the extent to which these findings match the predictions from constraint-based modeling. To determine the effect of the employed metabolic network model on the predictions, we perform a comparative analysis involving three state-of-the-art metabolic reconstructions of *A. thaliana*. In addition, we investigate three scenarios with respect to experimental findings on the ratios of the carboxylation and oxygenation reactions of Ribulose-1,5-bisphosphate carboxylase/oxygenase (RuBisCO). We demonstrate that the condition-dependent growth phenotypes of one of the engineered bypasses can be qualitatively reproduced by each reconstruction, particularly upon considering the additional constraints with respect to the ratio of fluxes for the RuBisCO reactions. Moreover, our results lend support for the hypothesis of a reduced photorespiration in the engineered plants, and indicate that specific changes in CO_2_ exchange as well as in the proxies for co-factor turnover are associated with the predicted growth increase in the engineered plants. We discuss our findings with respect to the structure of the used models, the modeling approaches taken, and the available experimental evidence. Our study sets the ground for investigating other strategies for increase of plant biomass by insertion of synthetic reactions.

## Introduction

The investigation and understanding of cell metabolism has recently experienced a paradigm shift largely propelled by the development of high-throughput methods. As a result, the classical pathway-centered approach has given way to a network-driven perspective, which considers the entire set of characterized biochemical reactions. This has led to the construction of genome-scale metabolic models (GEMs) for organisms from each of the three domains of life: archaea, bacteria, and eukarya (Schellenberger et al., 2010). While a GEM constitutes an organized and comprehensive system of knowledge about an organism, it also allows *in silico* analyses based on constraint-based methods, relying on the corresponding stoichiometric matrix representation and assumptions about cellular metabolism (e.g., operability in a steady state and reversibility of reactions). Flux balance analysis (FBA) has provided the basic framework for predicting growth and biomass yield as well as investigating reaction fluxes in a metabolic network (Varma and Palsson, 1993a,b; Lewis et al., 2012). Approaches in this, so-called, constraint-based framework usually invoke the steady-state assumption, whereby there is no change in the size of the metabolic pools. Therefore, without additional assumptions, constraint-based modeling framework does not account for changes over time [e.g., due to circadian rhythm, which in plants necessitates the switch between autotrophic and heterotrophic metabolism (Cheung et al., 2014)]. Extensions of this framework have subsequently allowed systematic investigations of network modifications (e.g., deletion and insertion of reactions from other species or under- and over-expression of gene products) directed at enhancing particular metabolic functions (Burgard et al., 2003; Pharkya et al., 2004; Ranganathan et al., 2010; Yang et al., 2011; Larhlimi et al., 2012; Lakshmanan et al., 2013).

A metabolic network that includes all known biochemical reactions of an organism may not be realistic in a particular cellular scenario (i.e., context), as mounting evidence shows that cells adapt their metabolism to the prevailing circumstance (e.g., external environment, developmental stage, cell type in multicellular organisms). In different cellular contexts, only a subset of reactions is typically active, which may lead to differences in biomass composition (Chang et al., 2011; Arnold and Nikoloski, 2014). Therefore, the shift toward reconstructing context-specific models of cell metabolism has become necessary to provide more accurate and biologically meaningful insights (Bordbar et al., 2014; Machado and Herrgård, 2014; Robaina Estévez and Nikoloski, 2014). This is of particular importance when tackling the physiology of multicellular organisms, not only to better understand tissue- or cell-specific metabolism, but also as a first step to reconstruct the metabolic network of an entire plant, whereby multiple specialized models are mutually interconnected (de Oliveira Dal’Molin et al., 2015).

Despite these recent developments, it remains questionable to what extent constraint-based methods and large-scale modeling can be used for devising metabolic engineering strategies in plants. The ideal approach to test whether or not modeling based on existing plant GEMs can provide insights into crop optimization and predict novel optimization strategies is to investigate the extent to which the predictions match observations from metabolic engineering experiments. Since photorespiratory bypasses have recently been successfully engineered and tested in the model plant *Arabidopsis* (Peterhansel et al., 2013b), they can be used to evaluate the capability of existing models to correctly predict novel synthetic engineering strategies. We, therefore, focus on the analysis of plant photorespiratory metabolism in the metabolic network context.

Photorespiratory metabolism and its experimentally investigated bypasses represent an excellent test case to investigate the potential of plant GEMs in plant metabolic engineering for two reasons. First, the existing GEMs in the model C_3_ plant *Arabidopsis thaliana* (and other photosynthetic organisms) include almost all components of the photorespiratory metabolism (Arnold and Nikoloski, 2013); therefore, they provide a suitable starting point for investigating the role of photorespiration in the network context. Second, photorespiratory metabolism does not operate in isolation, but shapes the energetics of photosynthesis, compartmental reductant exchange, nitrate assimilation, one-carbon (C_1_) metabolism, and redox signal transduction [for recent reviews, see Foyer et al. (2009) and Bauwe et al. (2012)]; therefore, investigating the effects of modulating this pathway necessitates adoption of a network perspective.

**Figure 1:**
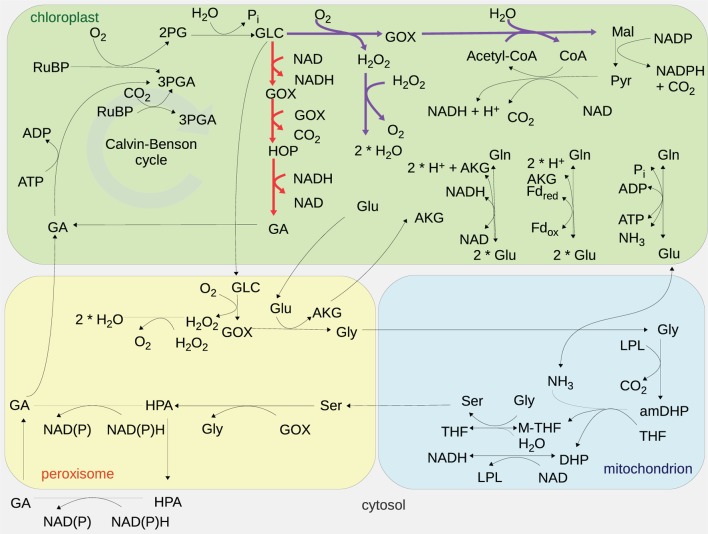
**Graphical representation of the modeled photorespiratory pathway and the two photorespiratory bypasses considered**. The Maier bypass is drawn in purple, while the Kebeish bypass is displayed in red. Cytosolic reactions of the photorespiratory pathway are not depicted since the models used only consider these reactions in the chloroplast, peroxisome, and mitochondria. The abbreviations are as follows: carbon dioxide (CO_2_), oxygen (O_2_), ribulose-1,5-bisphosphate (RuBP), 3-phosphoglycerate (3PGA), phosphoglycolate (2PG), glycolate (GLC), glyoxylate (GOX), hydrogen peroxide (H_2_O_2_), glutamate (Glu), glycine (Gly), α-ketoglutarate (AKG), serine (Ser), ammonia (NH_3_), 3-hyrdoxypyruvate (HPA), glycerate (GA), glutamine (Gln), 2-hydroxy-3-oxopropanoate (HOP), malate (Mal), pyruvate (Pyr), coenzyme A (CoA), acetyl-coenzyme A (acetyl-CoA), water (H_2_O), phosphate (Pi), lipoylprotein (LPL), aminomethyldihydrolipoylprotein (amDHP), tetrahydrofolate (THF), 5,10-methylenetetrahydrofolate (M-THF), dihydrolipoylprotein (DHP), oxidized nicotinamide adenine dinucleotide (NAD), reduced nicotinamide adenine dinucleotide (NADH), adenosine diphosphate (ADP), adenosine triphosphate (ATP), and ferredoxin (Fd).

The plant photorespiration pathway involves 12 reactions which are partitioned among three compartments, namely chloroplast, peroxisome, and mitochondrion, with bypasses in the cytosol (Figure 1) (Timm et al., 2008). Ribulose-1,5-bisphosphate carboxylase/oxygenase (RuBisCO) reacts with ribulose-1,5-­bisphosphate (RuBP), resulting in the enediol-enzyme complex that can in turn react with oxygen (O_2_), termed *oxygenation* (Calvin, 1954; Ogren and Bowes, 1971), or carbon dioxide (CO_2_), termed *carboxylation*. Upon carboxylation, one molecule of RuBP is transformed into two molecules of 3-phosphoglycerate. The oxygenation of RuBP yields one molecule of 3-phosphoglycerate and one molecule of phosphoglycolate. To mitigate the inhibition of the photosynthetic pathway by phosphoglycolate (Peterhansel et al., 2013b), this compound is recycled in a series of reactions comprising the photorespiratory pathway. For completeness, Table 1 includes the enzyme names, Enzyme Commission (EC) numbers, the corresponding biochemical reactions together with their compartmentation and reversibility, obtained from AraCyc 9.0 (Mueller et al., 2003). From the considered reactions, photorespiration can be regarded as uptake of O_2_ and phosphoglycolate-related evolution of CO_2_ and ammonia (NH_3_) (effectively refixed by the glutamine synthetase and the glutamate synthase).

**Table 1:** **Enzymes and reactions of the photorespiration including different compartmentation for *A. thaliana* (Foyer et al., 2009)**.

Enzyme name	EC	Enzymatic reaction	Compartment
Ribulose-1,5-bisphosphate carboxylase/oxygenase	4.1.1.39	RuBP + CO_2_ → 2 3PGA	(h)
		RuBP + O_2_ → 3PGA + 2PG	
Phosphoglycolate Phosphatase	3.1.3.18	2PG + H_2_O → GLC + Pi	(h)
Glycolate oxidase	1.1.3.15	GLC + O_2_ → GOX + H_2_O_2_	(p)
Catalase	1.11.1.6	2 H_2_O_2_ → 2 H_2_O + O_2_	(p)
Glycine transaminase	2.6.1.4	Glu + GOX → Gly + AKG	(p)
Glycine dehydrogenase (decarboxylating)	1.4.4.2	Gly + LPL → amDHP + CO_2_	(m)
Aminomethyltransferase	2.1.2.10	amDHP + THF → DHP + M-THF + NH_3_	(m)
Dihydrolipoyl dehydrogenase	1.8.1.4	DHP + NAD ↔ LPL + NADH	(m)
Glycine hydroxymethyltransferase	2.1.2.1	Gly + M-THF + H_2_O ↔ Ser + THF	(m)
Serine-glyoxylate transaminase	2.6.1.45	Ser + GOX → HPA + Gly	(p)
Glycerate dehydrogenase	1.1.1.29	HPA + NADH → GA + NAD	(p)
Hydroxypyruvate reductase	1.1.1.81	HPA + NAD(P)H → GA + NAD(P)	(p)
Glycerate kinase	2.7.1.31	GA + ATP → 3PGA + ADP	(h)
Glutamine synthetase	6.3.1.2	Glu + NH_3_ + ATP ↔ Gln + ADP + Pi	(h)
Glutamate synthase (ferrodoxin dependent)	1.4.7.1	2 Glu + Fd_ox_ ↔ Gln + AKG + Fd_red_ + 2 H^+^	(h,m)
Glutamate synthase (NADH-dependent)	1.4.1.14	2 Glu + NAD ↔ Gln + AKG + NADH + 2 H^+^	(h,m)
NADP-malic enzyme	1.1.1.40	Mal + NADP → Pyr + NADPH + CO_2_	(h)
Pyruvate dehydrogenase	1.2.4.1	Pyr + CoA + NAD → Acetyl-CoA + NADH + H^+^ + CO_2_	(h)

*The abbreviations of the compartment names in which the corresponding reaction takes place are as follows: (h), chloroplast; (m), mitochondrion; (p), peroxisome. The compound abbreviations are as follows: carbon dioxide (CO_2_), oxygen (O_2_), ribulose-1,5-bisphosphate (RuBP), 3-phosphoglycerate (3PGA), phosphoglycolate (2PG), glycolate (GLC), glyoxylate (GOX), hydrogen peroxide (H_2_O_2_), glutamate (Glu), glycine (Gly), α-ketoglutarate (AKG), serine (Ser), ammonia (NH_3_), 3-hyrdoxypyruvate (HPA), glycerate (GA), glutamine (Gln), water (H_2_O), phosphate (Pi), lipoylprotein (LPL), aminomethyldihydrolipoylprotein (amDHP), tetrahydrofolate (THF), 5,10-methylenetetrahydrofolate (M-THF), dihydrolipoylprotein (DHP), oxidized nicotinamide adenine dinucleotide (NAD), reduced nicotinamide adenine dinucleotide (NADH), adenosine diphosphate (ADP), adenosine triphosphate (ATP), ferredoxin (Fd). While EC 1.1.1.81 also operates in the cytosol (Timm et al., 2008), this function is included only in the peroxisome of the considered models; hence, the compartment given for this enzyme is (p)*.

Three photorespiratory bypasses have been experimentally investigated to date, recently reviewed for their benefits and energy balances (Peterhansel et al., 2013a,b). The bypasses of Kebeish et al. (2007) and Maier et al. (2012) were tested in *A. thaliana*, while the bypass of Carvalho et al. (2011) was examined in tobacco. Since GEMs for tobacco have not been assembled to date, we focus on investigating the first two bypasses (see Table 2 for the corresponding lists of reactions).

**Table 2:** **Enzymes and reactions of the photorespiratory bypasses of Kebeish et al. (2007) and Maier et al. (2012) in the chloroplast**.

Enzyme name	EC	Enzymatic reaction
**Kebeish bypass**		
Glycolate dehydrogenase	1.1.99.14	GLC + NAD → GOX + NADH
Tartronate semialdehyde carboxylase	4.1.1.47	2 GOX → HOP + CO_2_
2-hydroxy-3-oxopropionate reductase	1.1.1.60	HOP + NADH → GA + NAD
**Maier bypass**		
Glycolate oxidase	1.1.3.15	GLC + O_2_ → GOX + H_2_O_2_
Malate synthase	2.3.3.9	GOX + H_2_O + Acetyl-CoA → Mal + CoA
Catalase	1.11.1.16	2 H_2_O_2_ → O_2_ + 2 H_2_O

*Carbon dioxide (CO_2_), oxygen (O_2_), glycolate (GLC), glyoxylate (GOX), hydrogen peroxide (H_2_O_2_), glycerate (GA), 2-hydroxy-3-oxopropanoate (HOP), malate (Mal), pyruvate (Pyr), coenzyme A (CoA), acetyl-coenzyme A (acetyl-CoA), oxidized nicotinamide adenine dinucleotide (NAD), and reduced nicotinamide adenine dinucleotide (NADH)*.

The “Kebeish bypass” starts with glycolate and ends with glycerate by considering three reactions in the chloroplast, namely, glycolate dehydrogenase (EC 1.1.99.14), tartronate semialdehyde carboxylase (EC 4.1.1.47), taking glyoxylate and producing CO_2_ and 2-hydroxy-3-oxopropanoate, as well as 2-hydroxy-3-oxopropionate reductase (EC 1.1.1.60), transforming 2-hydroxy-3-oxopropanoate (and NADH, NADPH, and H^+^) into glycerate (and NAD, NADP) (Figure 1). The “Maier bypass” consists of a complete glycolate catabolic cycle, including glycolate oxidase (EC 1.1.3.15), malate synthase (EC 2.3.3.9), transforming glyoxylate (and Acetyl-CoA, H_2_O) into malate (and CoA), and catalase (EC 1.11.1.16) (Figure 1), extending earlier promising findings only using the latter two enzymes (Fahnenstich et al., 2008). Malate is decarboxylated to pyruvate by the chloroplastic NADP-malic enzyme (EC 1.1.1.40), while the chloroplastic pyruvate dehydrogenase (PDH, EC 1.2.4.1) converts pyruvate into acetyl-CoA, yielding NADH and one molecule of CO_2_. By interconversions in the glycolate cycle, one molecule of glycolate is converted into two molecules of CO_2_. Whereas the “Kebeish bypass” reintroduces three-fourth of the glycerate into Calvin–Benson cycle intermediates, the second bypass operates without recycling of 3PGA [and Calvin–Benson cycle intermediates are depleted (Peterhansel et al., 2013a)].

The aim of our study is to test the extent to which large-scale metabolic models reproduce the experimental observations on engineering the two photorespiratory bypasses. For this purpose, we evaluated three state-of-the-art *A. thaliana* models, and used them to assess the effect of model size and model quality on the accuracy of predictions. In addition, we tested different scenarios with respect to the increase predicted upon enforcing additional biochemical constraints on the ratio between the fluxes of the RuBisCO carboxylation and oxygenation reactions. To avoid introduction of bias about the directionality of the introduced bypass reactions due to inability to assess the subcellular concentration of the participating metabolites, we systematically investigated the effect of all combinations of reaction reversibilities. As our main contribution, we demonstrate that the predictions for the increase in biomass upon insertion of the photorespiratory bypasses are in accord with experimental evidence across the studied models only upon the consideration of additional biochemical constraints. Therefore, our findings indicate the need for inclusion of additional constraints when using large-scale plant models for the design of viable metabolic engineering strategies.

## Materials and Methods

### Models and Bypasses

The analysis is based on three large-scale models of *A. thaliana* metabolism: (1) a bottom-up (i.e., experiment-driven) reconstruction, where the operability of the incorporated reactions and metabolites is ensured by starting from well-documented and necessary biochemical pathways (Arnold and Nikoloski, 2014), (2) AraGEM, the first Arabidopsis genome-scale metabolic network, including primary metabolism of compartmentalized plant cells (de Oliveira Dal’Molin et al., 2010), and (3) a larger model that includes both primary and secondary metabolisms (Mintz-Oron et al., 2012). While the last two models come with a single biomass reaction, the smaller model of Arnold and Nikoloski considers three biomass reactions corresponding to biomass compositions that pertain to realistic and frequently examined scenarios: carbon-limiting, nitrogen-limiting, and optimal growth conditions (Kleessen et al., 2012). These biomass reactions were assembled by considering the composition of 1 g dry weight of Arabidopsis leaf under the respective conditions: growth under optimal condition reflects only, if any, light limitation under autotrophic conditions; nitrogen-limitation is based on a protocol that results in a mild but sustained restriction of growth upon restriction of nitrogen availability, while the carbon limitation is experimentally realized via short-day conditions (8:16 light–dark cycle) (Arnold and Nikoloski, 2014). The functional differences of these models have already been compared with respect to the ability to simulate photoautotrophic growth, number of blocked reactions, and flux coupling of reactions (Arnold and Nikoloski, 2014). This systematic comparative analysis indicated the suitability and added value of the bottom-up reconstruction, in which every reaction can carry flux, despite the greater degree of flux coupling, and, hence, more constrained flux space.

The photorespiratory bypasses were introduced by adding the corresponding reactions to the chloroplast of the three models: glycolate dehydrogenase, tartronate semialdehyde carboxylase, and 2-hydroxy-3-oxopropionate reductase for the Kebeish bypass; glycolate oxidase, malate synthase, and catalase for the Maier bypass. Since AraGEM does not contain glyoxylate or hydrogen peroxide in chloroplasts, we introduced the bypasses using the corresponding compounds from the cytosol (which is equivalent to introducing a transport reaction for allowing the exchange of these metabolites between the cytosol and chloroplast). The same approach was used for 2-hydroxy-3-oxopropanoate in the model by Mintz-Oron et al., which contains this metabolite only in the cytoplasm. Moreover, AraGEM and the model of Arnold and Nikoloski do not contain 2-hydroxy-3-oxopropanoate in any compartment. We, therefore, added the metabolite to the chloroplasts to allow introducing the Kebeish bypass. Note that this leads to a full coupling (i.e., fixed flux ratios) between the tartronate semialdehyde carboxylase and 2-hydroxy-3-oxopropionate reductase reactions in these models, since 2-hydroxy-3-oxopropanoate can only be produced and consumed by these reactions.

### Flux Balance Analysis

Constraint-based modeling investigates the solution space of feasible flux distributions $\nu \in {\mathbb{R}}^{n}$ for *n* biochemical reactions in a metabolic network that is assumed to operate in a (quasi) steady-state, i.e., the concentrations $x\in {\mathbb{R}}^{m}$ of the *m* metabolites in the network are constant (Varma and Palsson, 1993a,b; Bordbar et al., 2014). The steady-state condition can be written as $\frac{dx}{dt}=S\nu =0$, whereby the stoichiometric matrix $S\in {\mathbb{R}}^{m\times n}$ captures the stoichiometry of all reactions. Upper and lower boundaries of the flux vector *ν*_min_ ≤ *ν* ≤ *ν*_max_ further constrain the solution space and are used to model physiologically relevant scenarios (e.g., limit on nutrient import, reaction reversibility, and environmental conditions). The steady-state condition and the flux boundaries determine the solution space that usually contains infinitely many flux distributions, since the system of linear equations *Sv* = 0 is, in practice, underdetermined. To probe the functionality of the network, FBA (Varma and Palsson, 1993a,b; Bordbar et al., 2014) assumes that metabolic behavior is guided by some optimization principles (e.g., optimal biomass yield). Here, the objective is to maximize the flux through the biomass reaction, *ν*_biomass_, and the resulting optimization problem yields a linear program:

$$\text{max }z=\nu_{\text{biomass}}$$

(1) $$\begin{array}{l} \text{s.t.}\\ S\nu =0\\ \nu_{\text{min}}\leq \nu \leq \nu_{\text{max}}. \end{array}$$

### Flux Variability Analysis

The solution of the linear programing problem in Eq. 1, above, is the maximum flux value of the biomass reaction, denoted by z*. FVA allows determining the minimum and the maximum value of flux that a given reaction can carry while ensuring maximum flux through the biomass reaction. These values can be obtained by solving the following linear program for a given reaction *i*:

$$\text{max}\left(\text{min}\right)\nu_{i}$$

(2) $$\begin{array}{l} \text{s.t.}\\ S\nu =0\\ \nu_{\text{min}}\leq \nu \leq \nu_{\text{max}}\\ \nu_{\text{biomass}}=z^{*}. \end{array}$$

### Flux-Sum

At steady state, the net rate for metabolite consumption and production is zero, but its turnover is not. The flux-sum ϕ*_*i*_* of internal metabolite *i*, used as a proxy for metabolite turnover, is defined as sum over all reaction fluxes *v_*j*_* around metabolite *i*, $\phi_{i}=0.5\sum_{j}\left|S_{ij}\nu_{j}\right|$ (Chung and Lee, 2009). The flux-sum at the optimum biomass z* from Eq. 1 is determined by the linear program:

$$\text{max}\left(\text{min}\right)0.5*\sum_{j=1}^{n}\left|S_{ij}\nu_{j}\right|$$

(3) $$\begin{array}{l} \text{s.t.}\\ S\nu =0\\ \nu_{\text{min}}\leq \nu \leq \nu_{\text{max}}\\ \nu_{\text{biomass}}=z^{*}. \end{array}$$

### Implementation

The models were obtained from the respective publications in which they were first analyzed. All analyses were carried out with the help of the optimization platform TOMLAB version 8.1 using the CPLEX solver with default parameter for MATLAB R2015a. All used models with the irreversible variants of the introduced bypasses are provided as a Datasheet S1 in Supplementary Material. The flux-sum was implemented with TOMLAB ­version 8.1 using the SNOPT solver and default parameters.

## Results

Our analysis includes three scenarios to compare and contrast the predictions from the models of *A. thaliana* metabolism with respect to actual biomass increase upon insertion of the two photosynthetic bypasses. In Scenario A, we used the models with the default flux boundaries and employed FBA to determine the extent of the increase in the optimal flux through the biomass reaction upon introduction of the bypass reactions (see Materials and Methods). The predictions from Scenario A may not be realistic, since the optimal biomass may not correspond to a flux distribution in which the ratio of the fluxes of the carboxylation and the oxygenation reactions catalyzed by RuBisCO is physiologically plausible. To this end, we considered Scenarios B and C whereby the optimal flux through the biomass reaction is determined with the additional constraint that *ν*_carb_ _=_ ∈*ν*_oxy_, with ∈ = 4 and ∈ = 1.5, respectively, for those cases from Scenario A where increase in biomass was predicted. The values for the ratios were selected to match observations about the range for the rate of photorespiration estimated either from labeling experiments or from gas exchange data (Sharkey, 1988; Szecowka et al., 2013; Ma et al., 2014; Heise et al., 2015).

Since AraGEM and the model of Arnold and Nikoloski already include the glycolate oxidase, we introduced only the malate synthase and the catalase reaction in the chloroplast to simulate the Maier bypass. For both bypasses, we determined and compared the maximum biomass yield, obtained by FBA, with and without each of the bypasses.

In addition, by applying FVA (see Materials and Methods), we determined the interval of flux values that the reactions in the photorespiratory pathway (Table 1) can take at the optimum for each of the three scenarios. Comparison of the intervals allowed us to test the hypothesis that upon insertion of the bypass the photorespiratory flux is reduced, but not completely diverted, in comparison to the case without bypass. Validating the hypothesis would imply that the predictions from the large-scale models are in line with experimental observations suggesting a two- to fivefold reduction of photorespiratory flux (Peterhansel et al., 2013a) upon insertion of the considered bypasses. The results obtained from the three models under the different scenarios are summarized in Table 3.

**Table 3:** **Predicted percentage increase in biomass yield of the three models when introducing the Maier and Kebeish bypasses under the three analyzed scenarios**.

			Arnold and Nikoloski			AraGEM	Mintz-Oron
			optimal growth	C-limiting	N-limiting		
Scenario A	Maier bypass	irrev	0	0	0	0	1.0
		MS rev	0	0	0	0.6	1.0
		rev	0	0	0	0.6	1.0
	Kebeish bypass	irrev	0	0	0	0.3	0.4
		TS rev	0	0	0	0.3	0.4
		rev	0	0	0	0.3	0.8
Scenario B	Maier bypass	irrev	0	0	0	0	1.0
		MS rev	0	0	0	2.7	1.0
		Rev	0	0	0	2.7	1.0
	Kebeish bypass	irrev	6.2	6.2	6.3	1.1	0.4
		TS rev	6.2	6.2	6.3	1.1	0.4
		rev	6.2	6.2	6.3	1.1	0.8
Scenario C	Maier bypass	irrev	0	0	0.1	0	1.0
		MS rev	0	0	0.1	1.6	1.0
		Rev	0	0	0.1	1.6	1.0
	Kebeish bypass	irrev	3.0	3.0	4.0	0.1	0.4
		TS rev	3.0	3.0	4.0	0.1	0.4
		rev	3.0	3.0	4.0	0.1	0.8

*irrev indicates that all reactions of the bypass are considered irreversible; MS rev and TS rev indicate that only malate synthase and tartronate semialdehyde carboxylase, respectively, are considered reversible; rev indicates that all reactions of the bypass are considered reversible. This includes each configuration of reaction reversibilities, which leads to an increase in predicted growth with respect to a more constrained configuration*.

### Scenario A – No Constraints on the Flux Ratio of Carboxylation and Oxygenation Reactions

We first considered the core model of *A. thaliana* with the three biomass reactions for photoautotrophic growth: carbon-limiting, nitrogen-limiting, and optimal growth conditions (Arnold and Nikoloski, 2014). The carbon-limiting scenario pertains to short-day conditions, where energy efficiency of carbon fixation may be more limiting for growth than under long-day conditions. Incidentally, this is the condition under which the advantage of both investigated bypasses was detectable (Peterhansel et al., 2013b).

Under the assumption that all of the inserted reactions are irreversible, our results showed that no biomass increase could be predicted for any of the considered environments following the insertion of the Maier bypass. By contrast, by allowing the malate synthase to act as a reversible reaction, the model predicted a very small increase (< 0.03%) over the three growth conditions (Table S1 in Supplementary Material). Reversibility of the other two reactions (glycolate oxidase and catalase) does not affect the predicted biomass yield. This finding implies that the insertion of the Maier bypass in the model of Arnold and Nikoloski had negligible effect on biomass increase. The Kebeish bypass with any configuration of reversibilities of three individual reactions did not result in increase of biomass, similar to the Maier bypass scenario above (see Table S4 in Supplementary Material, increase <0.02%).

The AraGEM model with all irreversible reactions for the Maier bypass did not result in an increase in biomass. However, the insertion of all reversible reactions led to an increase of 0.6% for the single biomass reaction that the model includes. The FVA indicates that the malate synthase carries a negative (net) flux (Table S5 in Supplementary Material). The insertion of the Kebeish bypass resulted in an increase of 0.3%, see Table S6 in Supplementary Material. However, both results pertain to the unrealistic scenario in which the RuBisCO oxygenase practically does not carry flux, which was revealed by the FVA.

We next investigated the larger model of Mintz-Oron et al., which also includes the glycolate oxidase, but only in the cytoplasm, mitochondria, and peroxisome. Upon introducing the Maier bypass, the predicted increase in the optimal biomass yield was 1.0% with every combination of reaction reversibilities. By inspecting the results from FVA, we found that the increase in biomass yield was associated, as one would anticipate, to larger flux ranges. The only reaction that had a fixed value at the optimum biomass with and without inclusion of the bypass was glutamine synthetase; its flux was more than fivefold reduced, lending support for the hypothesis of reduced photorespiration by Peterhansel et al. (2013a). These findings were invariant over all combinations of reaction reversibilites (Table S2 in Supplementary Material).

Inserting the Kebeish bypass into the model of Mintz-Oron et al. resulted in 0.4% increase of biomass with all reactions considered irreversible. Allowing for reversibility of tartronate semialdehyde carboxylase and the other two reactions irreversible resulted in a maximum increase of 0.8%. Glutamine synthetase was, again, the only reaction that had a fixed value at the optimum biomass with and without inclusion of the bypass; its flux was reduced by more than 1.2-fold (Table S3 in Supplementary Material).

### Scenario B – Constrained Flux Ratio of Carboxylation and Oxygenation Reactions (3:2)

In this section, we repeated the analysis under the additional assumption that the flux ratio of RuBisCO carboxylase and oxygenase reactions was fixed to 3:2. The Maier bypass in the model of Arnold and Nikoloski did not predict an increase in biomass in any of the three environments. On the other hand, the Kebeish bypass resulted in a predicted biomass increase of 6.2% for optimal, 6.2% for carbon-limited, and 6.3% for nitrogen-limited growth conditions. We also inspected the change in flux variability ranges in the model upon insertion of the Kebeish bypass. As in Scenario A, the fluxes of the reactions in the photorespiratory pathway at the optimal biomass yield with the additional ratio constraint vary in a small range. Upon insertion of the bypass, the upper bounds for the photorespiratory flux were lowered by ~10% (Table S4 in Supplementary Material).

Scenario B with AraGEM resulted in biomass increase of 2.7% for the Maier bypass with all reversible reactions. Here too, we found that the increase in biomass yield was associated with a negative net flux for the malate synthase reaction (Table S5 in Supplementary Material). The increase for the Kebeish bypass was 1.1% with the three reversibilities of reactions in Table 3.

Scenario B with the model of Mintz-Oron et al. resulted in the same increase in biomass yield as in Scenario A, above, for the Maier bypass. Here too, we found that the increase in biomass yield was associated with larger flux ranges. The reaction catalyzed by glutamine synthetase was the only which showed a fixed value at the optimum biomass with and without inclusion of the bypass; its net flux shifted in the direction of synthesizing glutamine (more than fivefold decrease). As in Scenario A, these findings were invariant over all combinations of reaction reversibilites (Table S2 in Supplementary Material). The results for the Kebeish bypass were largely invariant in comparison to that of Scenario A (Table S3 in Supplementary Material).

### Scenario C – Constrained Flux Ratio of Carboxylation and Oxygenation Reactions (4:1)

In this section, we repeated the analysis under the assumption that the flux ratio of RuBisCO carboxylase and oxygenase reactions was fixed to 4:1. The model of Arnold and Nikoloski predicted an increase in biomass of 0.1% for all three reversibilities for the Maier bypass under nitrogen limiting growth condition. The Kebeish bypass resulted in an increase of 3.0% for optimal, 3.0% for carbon-limiting, and 3.9% for nitrogen-limiting growth conditions, accompanied by increase in flux variability bounds (Table S4 in Supplementary Material). AraGEM resulted in an increase of 0.1% with the Kebeish pathway and 1.6% for the Maier bypass, while the model of Mintz-Oron et al. resulted in the same predictions as in Scenario B for both bypasses (Tables S2, S3, S5, and S6 in Supplementary Material). As in Scenario B, above, glutamine synthase for the model of Arnold and Nikoloski shows a constant value at the optimum biomass upon the insertion of the Kebeish pathway, which is at least 20% smaller than the values at the optimum biomass for the wild type.

Our earlier analysis demonstrated that consistent predictions can only be made for the Kebeish pathway with the three models. We also showed that this was the case for the model of Arnold and Nikoloski with biomass functions for three different scenarios. Since this model was reconstructed entirely from experimental data, lacks any blocked reactions, and gives the best quantitative match between the predictions and observations from the bypasses, the remaining analyses will consider only this model with and without the Kebeish pathway. Specifically, in the following we focus on (1) biomass increase and (2) variability of CO_2_ uptake upon varying the carboxylation to oxygenation ratio as well as (3) the comparison of co-factor flux-sums (as proxies for turn-over) between the wild type and transformants (i.e., upon insertion of the bypass).

### Biomass Increase at Varying Values for the Carboxylation to Oxygenation Ratio

**Figure 2:**
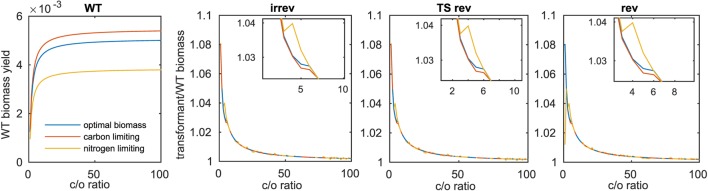
**Biomass increase at varying values for the carboxylation to oxygenation ratio**. For the three investigated biomass functions, **(A)** shows the optimal biomass yield obtained for varying carboxylation (c) to oxygenation (o) ratios, **(B–D)** show the fraction of transformant to wild-type biomass yield upon introduction of the Kebeish bypass with all reactions irreversible (irrev), the Kebeish bypass with reversible tartronate semialdehyde carboxylase (TS) and the Kebeish bypass with all reactions considered as reversible (rev).

Due to the observed differences in increase of biomass for the model of Arnold and Nikoloski upon insertion of the Kebeish bypass at two different fixed values of carboxylation to oxygenation ratios (i.e., 1.5 and 4), we expanded the range of investigated ratios to values between 1 and 100. This modeling scenario would account for differences in the internal CO_2_ concentration, resulting from the bypass insertion and manifesting itself in increases of carboxylation rate (Kebeish et al., 2007; Peterhansel et al., 2013a). Considering the wild type (Figure 2A), we observed, expectedly, an increase in biomass with the increase in the ratio between the carboxylation and oxygenation. The largest increase in biomass (~8%) for the transformants is observed at the smallest tested value for the ratio of 1. In each case, the largest difference between the three biomass functions was observed for ratios from 2.5 to 5, with an increase for the nitrogen-limiting biomass that outperforms the increase for the optimal and the carbon-limiting biomass functions. Interestingly, this region includes the previously tested value for the ratio of 4:1, which was derived from external gas exchange and labeling measurements [see the discussion in Heise et al. (2015)]. These observations held for the three cases of reversibilities considered in the Scenarios A–C, above (see Figures 2B–D and Table 3).

### Biomass Increase Is Associated with Differences in CO_2_ Uptake and Compartmentalization at Optimal Biomass Yields

In addition, we tested whether a higher CO_2_ concentration in the chloroplast could explain the growth increase observed by introduction of the Kebeish bypass (Kebeish et al., 2007). To this end, we analyzed the flux variability of CO_2_ import into the cytosol, the exchange of CO_2_ between the cytosol and the chloroplast, as well as the exchange of CO_2_ between the cytosol and the mitochondrion. We found that, upon insertion of the Kebeish bypass, the flux of CO_2_ into the chloroplast is reduced by 30% under Scenario B (carboxylation/oxygenation ratio of 3:2) and 6 to 9% for Scenario C (carboxylation/oxygenation ratio of 4:1) with all reversibilities examined above (Table 4 and Table S7 in Supplementary Material). From these results, we concluded that less CO_2_ needs to be imported from the cytosol, likely due to a higher CO_2_ concentration resulting from its release from the bypass. In line with a shift of CO_2_ release from mitochondria to the chloroplast, we observed a decrease in CO_2_ release from the mitochondrion into the cytosol. The uptake of CO_2_ from the environment into the cytosol, however, was increased. The latter result is in accordance with the experimentally determined increase in the apparent rate of CO_2_ assimilation in bypass transformants (Kebeish et al., 2007).

**Table 4:** **Minimum and maximum CO_2_ uptake and exchange flux at optimum biomass for wild type and transformant (T), including the irreversible Kebeish pathway for carboxylation to oxygenation (c/o) ratios of 3:2 and 4:1**.

	Range	c/o ratio 3:2			c/o ratio 4:1		
		Wild type	Transformant	Flux change in T compared to wild type (%)	Wild type	Transformant	Flux change in T compared to wild type (%)
A	Min	41.78	44.38	6.22	73.79	76.03	3.05
	Max	41.78	44.38	6.22	73.84	76.03	2.98
B	Min	57.85	40.74	−29.57	78.08	73.03	−6.47
	Max	57.85	41.46	−28.33	80.20	73.03	−8.94
C	Min	−20.79	−2.10	−89.91	−13.55	−4.06	−70.06
	Max	−20.65	−1.24	−94.00	−12.21	−3.02	−75.28

*A, flux range for CO_2_ uptake from environment into cytosol; B, CO_2_ exchange flux from cytosol to chloroplast; and C, ranges for CO_2_ exchange flux from cytosol to the mitochondrion. Results are presented for the biomass function derived from the optimal growth conditions (Arnold and Nikoloski, 2014)*.

### Biomass Increase Is Accompanied by Flux-Sum Differences in Co-Factors ATP, NADH, and NADPH

The flux-sum for a metabolite (see Materials and Methods) can be regarded as a measure for the overall flux through a metabolic pool at a feasible steady state (Chung and Lee, 2009). It has recently been applied in understanding changes in maize metabolism under different nitrogen conditions (Simons et al., 2014). Here, we were interested in comparing the range of the flux-sum values at the optimum biomass yield, while additionally enforcing either one of the previously considered values for the carboxylation-oxygenation ratios (3:2 and 4:1). We focused on interpreting the changes in compartment-specific flux-sum differences in co-factors ATP, NADH, and NADPH only in the cases where the ranges of their flux-sum in the transformants do not overlap with the ranges of the flux-sum in the wild type. In other words, we interpreted a change in turn-over that was valid in every possible steady state (with the imposed constraints). Therefore, we can conclude that the observed increase in the biomass is clearly associated with the difference in flux-sum values for these metabolites.

More specifically, we obtained a constant increase in flux-sum of ATP in the chloroplast and mitochondrion across all biomass fun­ctions and for both carboxylation to oxygenation ratios (Table S8 in Supplementary Material). In fact, the flux-sum of the chloroplastic ATP increased by at least 7% in Scenario B and 3.5% in Scenario C (% is determined from the maximum flux-sum in the wild type and the minimum flux-sum in the transformant). For the mitochondrial ATP, the increase was 85% in Scenario B and 143% for the carboxylation to oxygenation ratio of 4:1. Moreover, we observed that in the transformants the flux-sum of the peroxisomal NADH is fixed to 0 at the optimal biomass. The latter implies that NADH is not consumed nor produced by any of the modeled reactions in the peroxisome. For the remaining metabolites, while we observe changes in the ranges of the flux-sum, they overlap between the transformant and the wild type, and hence no conclusive statement can be made (Töpfer et al., 2015).

## Discussion

The two photorespiratory bypasses considered in our study have recently been investigated by Xin et al. (2015) through kinetic modeling by considering extensions to the model of Zhu et al. (2007). However, this approach cannot be employed to provide predictions about biomass yield, since the kinetic model only considered the core of plant carbon metabolism and, thus, lacks important biomass precursors, such as amino acids and cell wall components. Importantly, the kinetic modeling approach was based on the assumption that the photorespiratory pathway is not operational upon introduction of a bypass pathway, which has not been experimentally confirmed and, thus, precludes the possibility of testing the hypothesis of a reduced photorespiration. With respect to the rate of photosynthesis, the kinetic modeling predicted an increase following insertion of the Kebeish bypass but a decrease following insertion of the Maier bypass. However, gas exchange measurements indicated that the Maier bypass enhances the photosynthetic rate when calculated per mol chlorophyll (Maier et al., 2012). In addition, the predictions based on kinetic modeling depend on the type of enzyme kinetics, the values of the respective parameters, reversibility, and number and type of effectors. These are usually unknown, but will affect the predictions from the insertion of the bypasses and the reference state (i.e., fluxes and concentrations) used in the comparison.

In contrast to kinetic modeling, the constraint-based approach relies only on the assembled stoichiometry together with the assumption that the organism optimizes a particular objective – here biomass yield. While constraint-based modeling cannot make predictions about concentrations of intermediates without additional assumptions (Töpfer et al., 2015), using this approach one can make predictions of higher level phenotypes (e.g., biomass yield), as well as the fluxes associated with these phenotypes, based only on the metabolic reactions. To mitigate the effect of the model size and different reconstruction strategies on the predictions, we conducted a comparative analysis involving three state-of-the-art metabolic reconstructions of *A. thaliana*. We performed comparison with respect to increase in biomass yield and difference in the flux ranges for reactions involved in the photorespiratory pathway (Table 1) upon insertion of the bypasses. Moreover, unlike the kinetic modeling approach by Xin et al. (2015), we did not block photorespiratory flux, but instead left its flux unconstrained, which allowed predicting the effect of the bypasses on photorespiration with respect to optimal biomass yield. For instance, in Scenarios A and B for the model of Mintz-Oron et al., we found that the flux through the glutamine synthetase (which was constant at the optimum biomass with and without the bypass) was fivefold reduced upon introduction of the Maier bypass. Moreover, in Scenario B for the model of Arnold and Nikoloski, we found that the introduction of the Kebeish bypass lowered the upper bounds for the photorespiratory flux by 10%. Therefore, this constraint-based modeling approach allowed us to arrive at the prediction that the reduction of flux through the photorespiratory pathway, suggested by the experiments, indeed, holds.

Our results demonstrated that the constraint-based modeling approach predicts an increase in biomass upon insertion of the bypasses without the need for extensive model parameterization. Furthermore, this approach allowed us to test the effects of all possibilities for reaction reversibility in the bypass and the reactions involved in the photorespiratory pathway. However, we concluded that the qualitative match (i.e., increase in biomass yield of up to 6.22%) between the predictions and experimental evidence can be obtained only by further constraining the optimal states using experimental observations (namely, the ratio of RuBisCO carboxylation to oxygenation rates). Therefore, our findings indicate that predictions of metabolic engineering strategies are tightly bound to the reference state used, and bring to question approaches which attempt pathway engineering without considering reaction rates in the rest of the network.

In addition, our approach of simultaneous usage of *A. thaliana* metabolic models with different characteristics allows us to test the extent to which the metabolic engineering predictions may differ due to the intrinsic differences between the models. For instance, in the model of Arnold and Nikoloski, all reactions involving metabolic conversions (i.e., all reactions except those involved in maintenance, transport, import, and export processes, as well as biomass production) are annotated and there is experimental evidence for their occurrence in *A. thaliana*. By contrast, such evidence is missing for 21 and 37% of the annotated reactions in AraGEM and the model of Mintz-Oron et al. as a result of their (semi-)automated reconstruction using reaction databases. Moreover, and most importantly, the model of Arnold and Nikoloski alongside AraGEM does not include blocked reactions (i.e., reactions not carrying flux in any steady state). The percentage of blocked reactions in the model of Mintz-Oron et al. is 59%. Due to the inclusion of fewer reactions, the model of Arnold and Nikoloski is, however, less flexible (i.e., has smaller flux variability ranges and higher coupling of reactions) than AraGEM and the model of Mintz-Oron et al. In addition, the model of Arnold and Nikoloski predicts more efficient conversion of CO_2_ into biomass than AraGEM, at the cost of assimilating more photons. These increased levels of realism, discussed in detail in Arnold and Nikoloski (2014), may explain the better quantitative match between the predictions from the model of Arnold and Nikoloski upon placing physiologically meaningful constraints on the RuBisCO catalyzed reactions.

We attempted to link the increase in biomass to changes in several key molecular properties proposed to be associated with photorespiratory metabolism. To this end, we investigated the change in biomass increase as a function of an expanded range of values for the ratio of the RuBisCO carboxylation and oxygenation reactions. This allowed us to conclude that the increase in growth conferred by the bypasses may be due to the release of CO_2_ in chloroplasts and the resulting increase in internal CO_2_ concentration. This finding was further strengthened by simulating the changes in the CO_2_ transport fluxes between compartments as well as the uptake of CO_2_ from the environment, which is in line with the experimental evidence. Finally, we found changes in the flux-sum for ATP and NADH at optimum biomass, indicating that their pool sizes are associated with the predicted increase in biomass. Altogether, these molecular properties provide further evidence for the plausibility of the predictions from the constraint-based modeling of the photorespiratory bypasses in Arabidopsis.

Nevertheless, one question remains open: why is the predicted biomass yield much smaller than what was observed in experiments (e.g., ~17–20% for the Maier bypass [Maier et al., 2012)]? The reasons may very well be related to the quality of the underlying models, the modeling strategy taken, and the available experimental evidence: the considered models already incorporate some of the reactions that are integral to the analyzed bypasses. For instance, the model of Arnold and Nikoloski includes a catalase in the chloroplast, which is deemed essential for growth (i.e., its knock-out, by restricting the flux to 0, results in zero biomass yield). Therefore, the effect of inserting the remaining reactions of the bypass may be masked. In addition, experimental evidence indicates that the reference point (i.e., enzyme activity, metabolite levels) leading to biomass values used for gaging the increase may not correspond to the absolute optimum (i.e., optimum with no additional constraints) (Maier et al., 2012). Hence, the predicted optimal growth from the original (unmodified) plant GEMs may overestimate the actual growth rate of the wild-type plants, therefore partially masking the beneficial effect of introducing the bypasses. We have partly attempted to remedy the latter by enforcing particular flux ratios for the RuBisCO catalase and oxygenase; however, this is only one of many conceivable options. In addition, none of the experimental studies of the bypasses reported the growth rate of the plants, but instead the increase in biomass between wild type and the transgenic plant at a certain point of time. Therefore, the comparison between the growth rates (or yield) predicted by the constraint-based modeling framework cannot be directly compared with the end-point cumulative measurements from the experiments.

Further developments of this modeling framework may allow improvements in the accuracy of predictions by considering additional constraints from data (e.g., transcriptomics, proteomics, and metabolomics profiles) (Nikoloski et al., 2015). These could be complemented with easy-to-use computational tools for prediction and investigation of intervention strategies. Altogether, our study indicates that constraint-based modeling has indeed the potential to identify target reactions and pathways whose insertion could lead to increase in performance of plant species; however, our study indicates that the consideration of more physiologically meaningful constraints, rather than only simple optimization criteria, leads to predictions in line with existing experimental findings. Our study demonstrated that predictions across models of different sizes and levels of realism can provide additional reinforcement for the validity of plant-growth predictions from the constraint-based modeling framework.

## Author Contributions

GB and ZN designed the research. AK and GB performed the analyses. All authors interpreted the results and wrote the manuscript.

## Conflict of Interest Statement

The authors declare that the research was conducted in the absence of any commercial or financial relationships that could be construed as a potential conflict of interest.
